# Applications of Microwave Energy in Medicine

**DOI:** 10.3390/bios11040096

**Published:** 2021-03-26

**Authors:** Alexandra Gartshore, Matt Kidd, Lovleen Tina Joshi

**Affiliations:** 1 School of Biomedical Science, University of Plymouth, Plymouth PL4 8AA, UK; alexandra.gartshore@plymouth.ac.uk; 2 Emblation Microwave Ltd., Alloa, Scotland FK10 2HU, UK; matt.kidd@emblation.com

**Keywords:** microwaves, medicine, bacteria, ablation, tumours, diagnostics

## Abstract

Microwaves are a highly utilized electromagnetic wave, used across a range of industries including food processing, communications, in the development of novel medical treatments and biosensor diagnostics. Microwaves have known thermal interactions and theorized non-thermal interactions with living matter; however, there is significant debate as to the mechanisms of action behind these interactions and the potential benefits and limitations of their use. This review summarizes the current knowledge surrounding the implementation of microwave technologies within the medical industry.

## 1. Introduction

**Figure 1:**
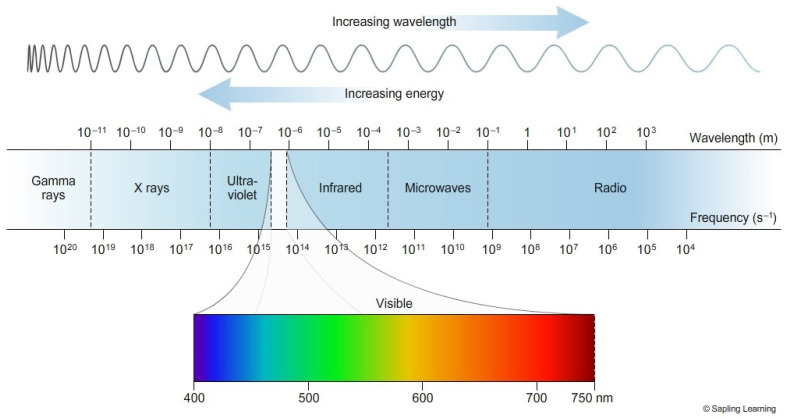
Electromagnetic spectrum. A depiction of the range of frequencies and wavelengths in the electromagnetic spectrum and the sub-ranges, as the wavelength increases the energy of the wave decreases [1].

**Figure 2:**
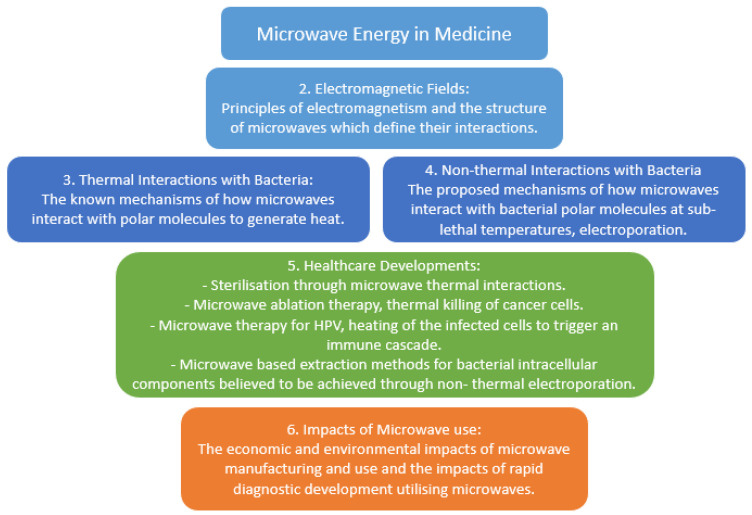
Summary of topics covered within microwave energy in medicine. Diagram depicts key themes described in this review.

Microwaves are a section of the electromagnetic (EM) spectrum (Figure 1; [1]): this spectrum ranges from radio waves to gamma rays. The EM spectrum can be expressed as frequency, which is measured in Hertz, wavelength and energy. Shorter waves with a higher value of energy such as ultraviolet are classed as ionizing as they generate sufficient energy to produce ions at a molecular level, causing damage to DNA and proteins. Whilst longer waves such as visible light are classified as non-ionizing; these can still cause thermal damage, however, this damage is not caused through ions. Microwaves are a type of electromagnetic radiation with free-space wavelengths ranging from 1 metre to 1 millimetre, with the frequency ranging between 300 MHz and 300 GHz, respectively [2]. The most common microwave frequency used is centred at approximately 2.45 GHz, which lies within the Industrial Scientific and Medical (ISM) radio band and is reserved for such purposes [3]. In recent years, microwave energy has been successfully exploited within medicine to treat diseases such as cancer and microbial infections via ablation therapy. There is now increased interest in using a range of microwave frequencies other than 2.45 GHz in the treatment of diseases; however, there is still limited understanding of the mechanisms of action of microwaves that induce biological changes in organisms. In this review, we focus primarily on microwave interactions at a cellular level with bacteria as model organisms. Herein we examine current literature regarding the functionality, current and prospective uses of microwave energy across a range of frequencies to demonstrate state of the art microwave advances in the medical industry (Figure 2) [4,5].

## 2. Electromagnetic Fields 

**Figure 3:**
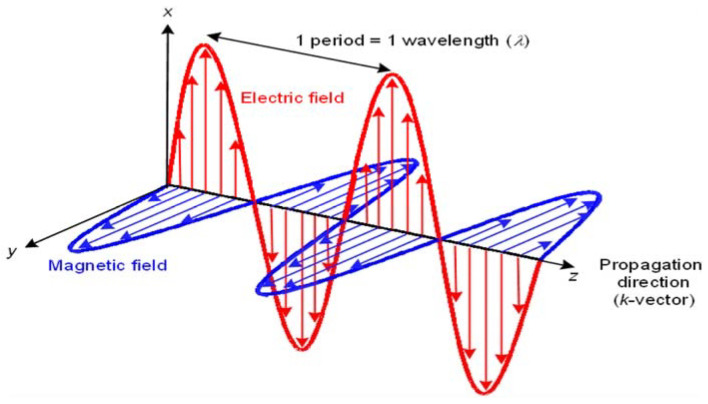
Electromagnetic wave diagram. Showing the direction of the wave, the direction and oscillation of the electric field and the direction and oscillation of the magnetic field. Each runs perpendicular to another; direction travelling along the x-axis, electric along the y-axis and magnetic along the z-axis [12].

An electromagnetic field consists of both a magnetic and an electric field produced by positively or negatively charged particles (Figure 3; [2]). An electric field is generated when particles gain a charge, either positive or negative via the transfer of electrons. If the electrically charged particles start to move, they produce an electric current; this current produces a magnetic field around the electric current. The electric field does not have to be moving to produce a magnetic field; if the charge of the electric field is fluctuating then a fluctuating magnetic field will be induced. Due to their coupled nature, if the correct balance is achieved, the fields can sustain each other and once sustained, an electromagnetic field emits directional electromagnetic waves as the fields fluctuate [6]. 

Both magnetic and electric fields are bound by laws of attraction which state that opposite charges always attract while ‘like’ charges repel. The strength of the attraction or repulsion is negatively proportional to the distance of the charges. One of the best examples of these laws is the chemical bonding between atoms via charged electrons and protons, these interactions can be described mathematically with Coulomb’s law [7]. Magnetic fields are produced by the presence of two charges that create field lines, where these lines intersect is described as poles, an example of this is the Earth’s North and South pole, and such magnetic charges can only exist as dipoles, not monopoles. Mathematical equations such as Maxwell’s equations prove the model for electromagnetism, furthermore, these equations describe how fluctuating electric and magnetic fields (Figure 3) travel at a constant speed [8]. Electromagnetic fields can act as waves and particles simultaneously; the waves travel outwards from their source and can move through a medium or a vacuum. In a vacuum, the wave travels at the speed of light, similarly, the air in our atmosphere is thin enough not to affect the propagation of the wave, however, when travelling through media the refractory index of the media will affect the movement of the waves. For media such as water, other factors alter the propagation; the high permittivity and electrical conductivity of water greatly increase the angle of refraction [9,10]. Another factor to consider is the microwaves ability to interact with polar molecules; water molecules are polar and so as the microwave passes through the water as a medium it also interacts with it. Polar molecules rotate when exposed to microwaves as they attempt to align with the waves’ fluctuating charges; the rotation produces heat and is the basis of microwave heating [11].

## 3. Thermal Interactions with Bacteria

**Figure 4:**
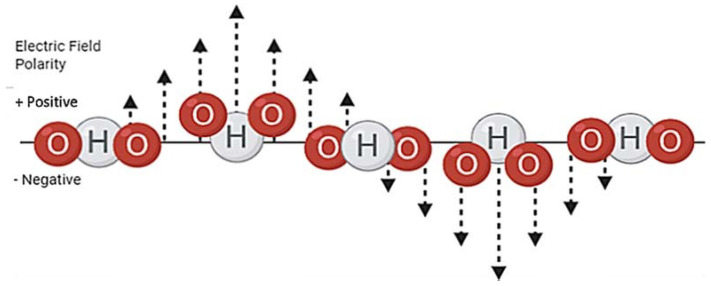
Water molecules rotation to align with an oscillating electric field. The electric field in a microwave oscillates between positive and negative polarity, the water molecules negative oxygen and positive hydrogen particles rotate to abide by the laws of attraction. This rotation generates thermal energy.

Microwaves are commonly used to heat food whilst reduce the microorganisms found within. Microwave heating reduces the number of microorganisms within food via direct thermal killing of cellular targets that render the bacteria either dead or inactive and therefore unable to replicate [13]. The microwave heating process relies on the interaction between polar molecules and the microwaves; in a 2.45 GHz microwave oven, the microwave frequency is strong enough to cause water molecules in the food to rotate at a speed that generates heat to cook food safely (Figure 4) [14]. 

Bacterial walls, capsules and the media in which the bacteria are cultured in contain polar molecules which will rotate and produce heat when exposed to microwaves [15,16]. Direct thermal killing results in the death of the bacterial cell by an increase in temperature which in turn severely damage the peptidoglycan cell wall. In *Staphylococcus aureus* the cell loses D-alanine from the teichoic acidswhich results in the cell’s inability to perform certain metabolic processes. Proteins are directly damaged by heating as the bonds holding them together are destroyed; this can damage enzymes and structural proteins that result in a loss of functionality [17,18,19]. Heat can also affect the integrity of many cellular aspects causing the cell to become inactive; in Gram negative bacteria the outer membrane is damaged by heat and becomes sensitive to lysozyme and hydrophobic antibiotics [20]. Due to the dependency microwave heating has with polar molecules, dried samples are not affected due to the lack of polar molecules, while those in the presence of water can reach lethal temperatures [21]. There is a debate that the non-thermal effects could contribute to the mechanism of destruction, as at sub-lethal temperatures enzyme activity is altered in *S. aureus* and in turn, could result in a change in bacterial functionality [22].

## 4. Non-Thermal Interactions with Bacteria

Electroporation is one of the non-thermal effects thought to be caused by microwave irradiation. In this concept, microwaves at sub-lethal temperatures induce the formation of pores in a cellular membrane due to their interaction with polar molecules. Although this is yet to be fully understood, current theories suggest that the polar molecules in the cell membrane rotate and create reversible pores; once the microwave is removed the pores close and return to the original structure [23,24,25,26]. These pores allow the cellular contents to leak outside, including substances such as DNA that are not normally able to cross the cell membrane. These released components from the cell are fully intact at sub-lethal temperatures and once purified can be used for further research [26]. Cells have shown to initially shrink after non-lethal microwave exposure, however, once the pores close, within 30 s the cell has been observed to return to its original size [22,27]. Utilising electroporation in the development of medical treatments and diagnostics is desirable due to its speed and low cost. Many researchers have found that electroporation is a viable tool for bacterial identification, DNA extraction and as a delivery system for molecules into cells. Keydevelopments and methods are discussed below.

## 5. Healthcare Developments 

Microwaves have a wide variety of uses within and outside of healthcare. In the 1950s microwaves were developed for communication and later for navigation with the use of relay links and satellites, exploiting their properties as electromagnetic waves [28,29]. However, it is the interactions with polar molecules in substances that have led to the development of many uses within the healthcare setting. The thermal interactions have been adapted for sterilization, sample preparation and ablation of cancerous cells; the rapid heating and thermal killing induced by the microwave interactions with polar molecules make this viable and effective. While the non-thermal interactions with polar molecules are being exploited for microwave imaging and extraction of intracellular components for rapid diagnostics, the permittivity of the wave and mechanism of electroporation make these uses practical although not yet perfected [30,31]. 

Sterilisation via microwave exposure has been developed through microwave heating and a series of treatments. Microwaves between 225 MHz to 100 GHz are primarily suited for sterilisation: the main microwaves used for heat sterilization of food in this range are at 2.45 GHz [32]. 2.45 GHz microwaves have also proven to be able to sterilise glass and plastics in as little as 180 s; this too requires the presence of water within the microwave to act as a heat sink and interact with the electromagnetic waves. This method of sterilisation can be used on both laboratory and medical equipment in place of an autoclave [33,34]. 

Another use of thermal interactions is Microwave Metal Sample preparation through microwave digestion. Microwave digestion is a technique to dissolve heavy metals in the presence of organic matter. This process exposes samples to strong acids and then raises the temperature using focused microwaves. This method can be used for environmental samples to measure contaminants that could affect human health, such as lead. For samples of soil to be analysed, they must be transformed into liquid samples through microwave digestion. The samples can then be analysed via Inductively coupled plasma mass spectrometry (ICP-MS) for trace metals or flame atomic absorption spectroscopy (FAAS) for major elements [35]. Microwave digestion and the subsequent spectrophotometry can be used to analyse trace metals in human tissue such as hair, nails and gallstones. Gallstones have trace amounts of metal that are associated with bilirubinate and black pigmented gallstones, thus by determining the amounts and variety of metals within gallstones their origin of these metals may be determined [36,37]. 

**Figure 5:**
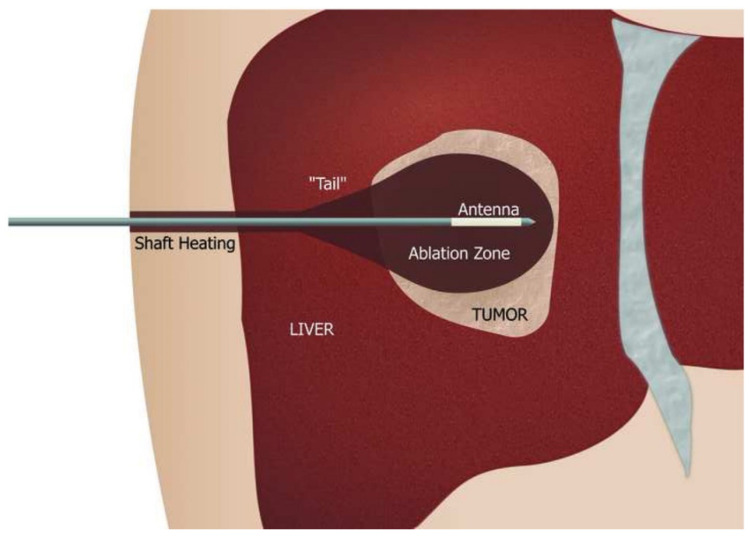
Microwave ablation schematic. The microwave antenna is made up of an applicator shaft that has temperature monitoring to combat shaft heating. Shaft heating occurs due to the reflection of the microwave and a cooling system is installed along the shaft to prevent burning. The antenna is most often needle-shaped but can have a variety of designs including monopole, dipole, and slotted antennas. The antenna design determines the tissue heating pattern and therefore impacts the ablation zone size and shape [39,40].

Ablation therapy is a state of the art treatment and destructive tool for abnormal tissues via heating by radio waves or microwaves [38]. Microwave ablation is a method of thermal tumour ablation where tumours are heated to damage the structure and proteins of the cell. As tumours have a higher water content than healthy tissues, the microwaves induce rapid heating via interacting with the polar water molecules within the tumour cells. Due to the microwaves ability to propagate through media, they can pass through and heat various tissues making it an applicable thermal ablation therapy for a variety of tissues [4,5,39,40]. Microwave ablation therapy for hepatocellular carcinoma is being viewed as a potential first line treatment for tumours on the liver surface, in which the tumour is exposed to a 2.45 GHz microwave with a wattage of 80–100 (Figure 5) [5,41]. Microwave ablation therapy has not only been successful within the liver, clinical studies have also shown complete ablation of tumours within the kidneys, lungs and bone; indeed, the majority of studies have resulted in no recurrent tumours [42]. Despite the success of microwave ablation, the use of this treatment is not as widely practised as expected, this is partly due to the expertise and equipment needed to perform the therapy, alongside competing for ablation therapies, such as radiofrequency ablation therapy [43,44,45]. Radiofrequency ablation yields similar successful tumour ablation results, however, there are advantages to microwave ablation, microwaves produce higher temperatures with shorter ablation times and a smaller heat sink effect [46,47]. Ablation therapy is a key development in the treatment of tumours, allowing their destruction without the need for surgery; the increased usage of both ablation techniques could reduce the cost and time needed to treat small tumours [48,49].

Microwaves can play a role in both the detection and treatment of breast cancer. Microwave imaging has been researched as an alternative to X-rays and ultrasound screening which have a variety of limitations [5,50]. Microwave imaging is an appropriate alternative as it is a low cost, harmless and potentially easier to perform compared to current methods with high sensitivity. Imaging techniques rely on the knowledge of the permittivity and conductivity of malignant, benign and healthy breast tissue. Due to the higher water content in cancer cells the dielectric properties of the tissue differs when exposed to microwaves [51]. Despite the nearly 40 years of research into microwave breast imaging, there are still many limitations that prevent a commercially available device that include inappropriate algorithms and sensors; however, with wider clinical trials a viable microwave imaging system may be feasible in the near future [39,40]. Microwave ablation has also been trialled for the treatment of breast cancer and so far has proved to be successful in thermal ablation [52,53].

Other than ablation and rapid heating, treatments for damaged and keratinized cells have been researched and developed with controlled heating. The Swift^®^ microwave is an approved state of the art treatment for plantar warts caused by the human papillomavirus (HPV). Directly exposing the wart to 8 GHz microwaves at 8 Watts for 2 s causes the wave to interact with the keratinized skin and results in controlled heating of the tissue. There is also the suggestion that microwaves enhance the cross-presentation of dendritic cells that are key for the immune defence against HPV [54,55]. A similar method has been developed for the potential treatment of actinic keratosis (AK). The Swift 8GHz^®^ microwave is used to expose the ulceration to 4 W for hyperkeratotic AK or 3 Watts for nonhyperkeratotic AK for 3 s repeated in triplicate with a 20-s time gap between pulses. This treatment has resulted in the clearance of actinic keratosis with brief pain and minimal long term adverse side effects in 90% of applied sites [56]. Following this, the use of microwaves to treat benign cancerous and precancerous lesions caused by high-risk HPV was investigated. High-risk HPV results in an increased expression of 2 viral oncoproteins; E6 and E7. When *in vitro-* grown tumours were exposed to microwaves for 10 s both tumour cell death and a reduction in E6 and E7 in the treated zone and transition zone were observed. This reduction suggests that microwave interactions can reverse the cancerous phenotype caused by HPV; and that once an effective and proven method is developed it could provide a less invasive treatment for HPV benign tumours [57,58].

State of the art microwave-based molecular diagnostics that incorporate the non-thermal effects of microwaves are currently under development to tackle the delays and complexities of current methods; these include microwave assisted metal enhanced fluorescence and nanotube assisted microwave electroporation. Microwave assisted metal enhanced fluorescence (MAMEF) is a rapid diagnostic method being developed to detect bacterial infections at point of care. MAMEF combines the use of silver nanoparticles deposited on microscope slides which are impregnated with anchored DNA sequences specific to the target sequence- such as a bacterial species [59,60]. Low power microwave heating kinetically accelerates the hybridisation of the target DNA and a fluorescent DNA target (usually a conserved region of bacterial genomic DNA) is excited and detected by the process. This method is currently crude and not yet manufactured for wider laboratory use [61]. If commercialized appropriately and manufactured into an integrated device, MAMEF would be a useful point of care diagnostic due to its speed, specificity, low cost and simplicity [62]. 

Lyse-It is advertised as a rapid “single step” process that lyses cells and causes DNA/RNA fragmentation. It uses a glass slide with gold nanolayers deposited in a “bow-Tie” shape. Using a sticky silicone isolator (Sigma Aldrich), the sample is placed on the slide in the centre of the gold bow and microwaved in the centre of a conventional 2.45 GHz microwave oven for lysis. The theory is that the gold focuses the microwaves to release the fragmented DNA [63]. This method of lysing DNA is much quicker than the current diagnostic DNA extraction methods and could be incorporated into point of care diagnostics. However, crucially there are currently no benefits in sensitivity as the samples are contaminated by the microwaved gold, and currently, due to the cost of the Lyse It Slides this method is not yet suitable to be used as a common DNA extraction method within diagnostic laboratories [64,65]. 

MAMEF and Lyse-It are not the only devices exploiting the proposed non-thermal effects of microwaves as a method of DNA extraction for molecular diagnostics. Methods using microcentrifuge tubes as an alternative to the expensive Lyse It slides are being researched, however, these processes are still somewhat time-consuming due to the required centrifuge and wash procedures [66]. Development of other rapid diagnostic devices that utilize microwave-based DNA extraction and fragmentation as a first step to sample processing is in the early stages of development [67]. 

Microwaves are also being used in DNA extraction outside of a healthcare setting, in environmental samples such as sediment and soil; these ecosystems are hard to culture due to the diversity of organisms and growth conditions, therefore genetic typing is the primary form of identification. There are limitations with current enzymatic lysis methods as the high molecular weight genomic DNA required for further research needs to be relatively pure, whereas the DNA from environmental samples have a variety of contaminants. By implementing a microwave-based thermal shock lysis method in which environmental samples are exposed to 2.45 GHz, 600–700 W microwaves for 45 s, a relatively large amount of good quality DNA (20–23 kb) can be extracted.In a sample size of 300 μL of activated sludge, up to 50 μg of DNA was extracted via microwave thermal shock compared to the 30 μg extracted via enzyme-based protocols. After the lysis of the environmental samples, appropriate washing and amplification can be performed for the identification of the 16S ribosomal gene. The same thermal shock method can be used to extract RNA, followed by an alternative suitable washing and amplification step that can be used for further identification of the 16S rRNA gene. This method of lysis is not only cost-effective and quicker at identifying environmental samples but could be developed and implemented to detect infectious microorganisms within stool samples in patients in a healthcare setting once these methods have been proven to be robust [68,69].

**Figure 6:**
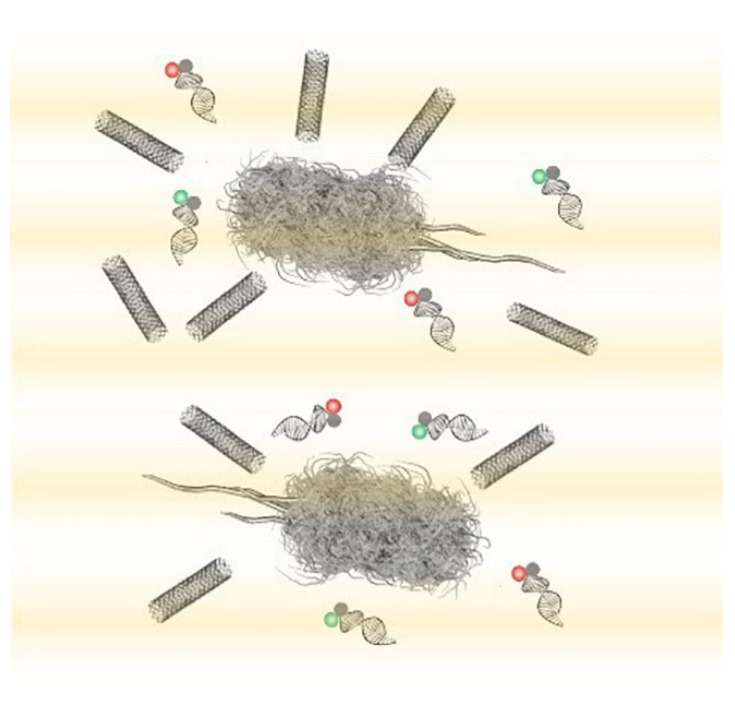
Nanotube assisted microwave electroporation (NAME) for single-cell pathogen identification. Multiwall carbon nanotubes induce localized electroporation for the delivery of multicolour double-stranded molecular probes for multiplex 16S rRNA detection [71].

Diagnostic and identification methods that do not rely on the extraction of intracellular components have also been researched. Nanotube assisted microwave electroporation (NAME) is a method that utilises electroporation; NAME however aims for visual identification rather than DNA extraction [70]. This technique can be applied to not only extract the contents of a bacterial cell but to act as a transport system to deliver molecules such as biosensors into the cells (Figure 6). In this method, carbon nanotubes are used as an antenna for coupling microwave energy; this localizes the electromagnetic field that induces bacterial electroporation in the cellular wall to the areas around the nanotube. This enhanced electroporation to specific areas (caused by the nanotubes) allows for the delivery of intracellular probes that consist of double stranded nucleic acid-targeting specific bacterial 16S rRNA and fluorophores, enabling the identification of *Escherichia coli*, *Klebsiella pneumoniae* and *Pseudomonas aeruginosa* for example at the single-cell level. An individualized probe was designed for each bacterial species. Bacterial samples of *E. coli*, *K. pneumoniae* and *P. aeruginosa,* after appropriate washing steps and the addition of the nanotube solution, were exposed to a 2.45 GHz microwave for 10 s, enabling electroporation and probe delivery. Once the probe was delivered into the bacterial cell via NAME the samples were mounted onto glass slides for observation under a fluorescent microscope; fluorescent cells were accurately identified. This delivery and identification method can be used directly on samples, and the whole process from the initial sample to microscopic identification can take as little as 30 min. NAME can identify pathogens such as those mentioned previously at a single cell level enabling accurate quantification via cell counts of fluorescent cells under the microscope, however further instrument development is required before this method can be clinically evaluated [71,72].

When comparing the state of the art microwave based DNA extraction methods with current commercially available DNA extraction kits, the overall opinion is that microwave methods are more efficient, cost-effective and simpler, so could be implemented without the need for specialised training [63,73]. The quantity and quality of DNA extracted by both techniques appear to be similar for cultured samples, while microwave techniques on specific samples such as blood can have varying results. If techniques and instruments are appropriately developed, microwaves have significant potential to enhance the rapidity and development of point of care diagnostic devices, for example, to tackle global healthcare challenges such as antimicrobial resistance [74]. The future perspectives of microwave use throughout the medical industry are increasingly positive. Microwave ablation has been approved as an effective treatment for a range of cancers with promising results to be implemented for use. If the development of methods and equipment continue then this could be a cost effective, minimally invasive method of treating a variety of sized and shaped tumours [40,75]. Although positive, the development of such techniques is not without challenges, controlling the direction and reflection of the wave is important in all treatments to not damage healthy tissue as well as the size of the delivery antennas [76]. Utilising microwaves for diagnosing breast cancer needs further research to refine the understanding of the dielectric properties of cancerous tissues and the required equipment. If these developments are made, microwave imaging could be pain-free, harmless and quicker than current screening methods such as mammography and X-ray [77]. 

## 6. Wider Impacts of Microwave Use

The impact of microwaves is widespread across medicine and has clear economic benefits; for example, the cost of cancer treatments could be reduced by thousands of dollars per person [78]. Another example is microwave sterilisation which, when compared to chemical sterilisation for medical equipment, has a variety of economic and environmental impacts. The production of microwaves responsible for sterilisation is expensive but is a one-time cost other than routine equipment maintenance and the running cost of electricity needed for microwave sterilisation is also not small, however, environmental impact is limited in correlation to the production and power supply of the microwaves. Chemicals for sterilisation are cheaper to purchase in small quantities, however, this is no more expensive than microwave use on a larger scale. Moreover, the environmental impacts are high throughout the manufacturing process, use and disposal of the chemicals [79,80]. 

The same economic and environmental effects of microwave use can be applied throughout all microwave developments. The cost of producing the microwave is high due to the resources, equipment and power required and therefore the environmental impact is high due to these resources being often non-renewable. The disposal of microwaves is one of the greatest sources of environmental impacts in the process if disposed of incorrectly, however, due to the Waste Electrical and Electronic Equipment recycling (WEEE) directives. a wide array of microwaves are recycled safely [81]. The environmental and economic impacts created from microwaves and point of care technology is always changing and generally reducing due to advances in consumer electric drives; as new technology is developed the parties involved will be actively attempting to reduce their environmental footprint and act sustainably [82].

## 7. Conclusions

Microwaves are a versatile electromagnetic wave with a variety of uses within and beyond medicine (Table 1). Currently, 2.45 GHz microwaves are primarily used for heating and sterilisation within the food industry and the thermal methods of this process are relatively well known. These microwaves have also been adapted for medical treatments, microwave ablation therapy is available for a variety of cancers and reduces both the cost and length of treatment. The 8 GHz microwave frequency has recently been utilised for the treatment of viruses and reduces the need for antimicrobial drugs. There are many areas in which microwaves are in development within medicine both in diagnostics and treatments, however, despite all these advancements in the uses of microwaves, the knowledge behind the mechanisms as well as the impact microwaves have are limited. There is no clear mechanism behind the non-lethal interactions between microwaves and bacteria due to the limited control over the temperature and direction of microwaves there are still many risks involved with current treatments, as microwaves interact with all polar molecules. The mechanism is not the only knowledge gap that needs to be filled, limited studies regarding the environmental and economic impacts of microwave use hinder the appropriate development of microwave technology. Despite these limitations, the diverse applicability to human healthcare will improve the equality and longevity of life for many, with a reduced burden on the economy.

## Figures and Tables

**Table 1:** Summary of Microwave Applications in Medicine.

Microwave Energy Application	Method	Example(s)	Ref.
Sterilisation	Thermal Energy. 225 MHz to 100 GHz; 2.45 GHz	Food, Glass, Plastics	[32,33,34]
Heavy Metal Digestion	Thermal Energy 2.45 GHz	Metals, Gallstones	[3,4,5,6,7,8,9,10,11,12,13,14,15,16,17,18,19,20,21,22,23,24,25,26,27,28,29,30,31,32,33,34,35,36,37]
Ablation Therapy	Thermal Energy 8 GHz, ~2.45 GHz	Oncogenic Tumors, Keratinised cell, Plantar Warts (HPV)	[39,40,41,43,44,45,54,55,56,57]
Diagnostics	Non-Thermal Energy Microwave Accelerated Metal Enhanced Fluorescence (MAMEF) 2.45 GHz	Bacterial pathogens	[59,60,61,62,63]
Lysis	Thermal Energy 2.45 GHz	Bacterial Pathogens	[64,65]
Electroporation	Non-Thermal Energy 2.45 GHz	Bacteria at single-cell level	[70]

## Data Availability

Not applicable.
